# Oral cannabidiol (CBD) as add-on to paracetamol for painful chronic osteoarthritis of the knee: a randomized, double-blind, placebo-controlled clinical trial

**DOI:** 10.1016/j.lanepe.2023.100777

**Published:** 2023-11-10

**Authors:** Sibylle Pramhas, Teresa Thalhammer, Sebastian Terner, Daniel Pickelsberger, Andreas Gleiss, Sabine Sator, Hans G. Kress

**Affiliations:** a Department of Special Anaesthesia and Pain Therapy, Medical University of Vienna, Waehringer Guertel 18-20, Vienna 1090, Austria; b Center for Medical Data Science, Medical University of Vienna, Spitalgasse 23, Vienna 1090, Austria

**Keywords:** Cannabidiol, Osteoarthritis of the knee, Pain

## Abstract

**Background:**

Painful knee osteoarthritis (KOA) is common, pharmacological treatment, however, is often hampered by limited tolerability. Cannabidiol, which preclinically showed anti-inflammatory, analgesic activity, could supplement established analgesics, but robust clinical trials are lacking. The aim of our study was to investigate the effects of oral high-dose CBD administered over 8 weeks on pain, function and patient global assessment as an add-on to continued paracetamol in chronic symptomatic KOA.

**Methods:**

Prospective, randomized, placebo-controlled, double-blind, parallel-group study. Single center, Outpatient Clinic, Department of Special Anaesthesia and Pain Therapy at Medical University of Vienna, Austria. Eligibility criteria included: age: 18–98 years; painful KOA; score ≥5 on the pain subscale of the Western Ontario and McMasters Universities Osteoarthritis (WOMAC) Index; KOA confirmed by imaging. Participants were on continued dosage of paracetamol 3 g/d and randomly assigned by web-based software 1:1 to oral cannabidiol 600 mg/d (n = 43) or placebo (n = 43). Study period: 8 weeks. Primary outcome: Change in WOMAC pain subscale scores (0 = no pain, 10 = worst possible pain) from baseline to week 8 of treatment. Trial Registration: ClinicalTrials.gov Identifier: NCT04607603. Trial is completed.

**Findings:**

The trial was conducted from October 1, 2020 to March 29, 2022. 159 patients screened, 86 randomized. Among 86 participants (mean age, 62.8 [SD 20.3] years; 60 females [69.8%]), 58 (67.4%) completed the trial. Mean baseline WOMAC pain subscale was 6.0 ± 1.1. Analysis: Intention-to-treat principal. Mean reduction in WOMAC pain subscale was 2.5 (95% CI: 1.8–3.3) in the cannabidiol group and 2.4 (95% CI: 1.7–3.2) in the placebo group with no significant group difference (p = 0.80). Adverse events were significantly more frequent with cannabidiol (cannabidiol: 135 [56%]; placebo: 105 [44%]) (p = 0.008). Rise above baseline of liver aminotransferases and gamma-glutamyltransferase was significantly more common in the cannabidiol (n = 15) than the placebo group (n = 5) (p = 0.02).

**Interpretation:**

In KOA patients, oral high-dose add-on cannabidiol had no additional analgesic effect compared to adding placebo to continued paracetamol. Our results do not support the use of cannabidiol as an analgesic supplement in KOA.

**Funding:**

Trigal Pharma GmbH.

**Research in context**

**Evidence before this study**

We conducted a search on PubMed up to September 21, 2023 using the search terms “cannabidiol”, “pain” and “randomized clinical trial”. This search yielded two previous randomized, placebo-controlled double-blind clinical trials in acute pain (CANBACK trial in acute low back pain; Cannabidiol for postoperative pain after arthroscopic rotator cuff repair) and two in chronic pain (Cannabidiol in hand osteoarthritis and psoriatic arthritis; Cannabidiol in peripheral neuropathic pain). Furthermore, two randomized placebo-controlled double-blind clinical trials in pain models in healthy volunteers (CANAB I and CANAB II) were identified.

In the CANBACK trial, patients with acute non-traumatic low back pain received a single-dose of 400 mg oral cannabidiol, which did not reduce pain scores significantly as compared to placebo. The trial in postoperative pain after rotator cuff repair investigated buccally absorbed cannabidiol at a dosage of 75 mg and 150 mg per day for 14 days postoperatively. A significant difference in VAS pain score was only observed on day 1. The clinical trial in hand osteoarthritis and psoriatic arthritis evaluated analgesic effects of 20–30 mg synthetic CBD per day versus placebo over a period of 12 weeks. No significant effects of CBD on pain intensity were detected. The trial in peripheral neuropathic pain applied a dosage of 30–50 mg CBD per day over a period of 8 weeks. No significant reduction of pain level as compared to placebo was reported.

Both the CANAB I (acute pain model, intradermal electrical stimulation) and CANAB II (acute pain model, intradermal electrical stimulation combined with opioid-induced hyperalgesia provoked by remifentanil infusion) applied a high single oral dose of 1600 mg CBD. Both CANAB I and CANAB II did not show any relevant effect of a high single dose of CBD on pain ratings.

**Added value of this study**

All previous trials on the analgesic effects of CBD on pain in humans either utilized doses far below those applied in the clinical trials that demonstrated its antiepileptic efficacy, or even only applied a single dose of CBD. Therefore, it remained unclear whether the negative results may have been due to under-dosing and/or a too short duration of application.

The added value of the current study consists in providing solid information on the analgesic potential of cannabidiol in a common and defined chronic pain condition, when applied over a prolonged period of time at daily oral doses similar to those utilized in previous positive clinical trials on epilepsy. In accordance with all previous trials in humans no analgesic effect of CBD could be observed.

**Implications of all the available evidence**

All available evidence from randomized placebo-controlled clinical trials and from randomized placebo-controlled trials in healthy volunteers, points against a significant analgesic potential of cannabidiol in humans.

This is particularly relevant, as cannabidiol is currently being marketed and recommended for pain relief by some suppliers without adequate evidence. The available data so far do not support the use of cannabidiol as an analgesic.

## Introduction

Symptomatic chronic osteoarthritis of the knee (KOA) is common in elderly adults (prevalence 9.5%, age 63–94 years) and accounts for significant disability and pain in these often multimorbid individuals.^1^

Systemic pharmacological treatments of painful chronic KOA are limited by contraindications and tolerability concerns, reflected by inconsistency of international guidelines on the use of non-steroidal anti-inflammatory drugs (NSAID), paracetamol and opioids.^2^^,^^3^

Therefore, the systematic investigation of oral cannabidiol (CBD) as a promising additional pharmacological option is clinically relevant.

A recent meta-analysis reported a small but significant antinociceptive effect of CBD in murine models of injury-related/persistent pain.^4^ CBD also improved pain-related behavior in two randomized, placebo-controlled, double-blind studies in spontaneous canine osteoarthritis.^5^^,^^6^ Based on such experimental data alone, CBD has immediately been touted for similar pain conditions in humans,^7^ although clinical evidence from rigorous, randomized, controlled trials on its analgesic efficacy is lacking.^7^, ^8^, ^9^

Two randomized placebo-controlled clinical trials have investigated the analgesic potential of CBD in acute pain. The CANBACK trial evaluated efficacy of single-dose oral CBD (400 mg) as an add-on to standard analgesics in acute non-traumatic low back pain. Here, decrease in verbal numeric rating scale was not superior to placebo in the CBD group.^10^ The single-dose application, however, limits the conclusive interpretation of study results. Alaia et al. investigated buccally absorbed CBD for pain control after arthroscopic rotator cuff repair. Low doses of CBD (75 mg or 150 mg per day) were utilized for 14 days postoperatively. Only on day 1, the visual analogue scale (VAS) score of the CBD group was significantly lower than in the placebo group.^11^

Two randomized placebo-controlled trials evaluated the analgesic effect of a single dose of CBD in a model of acute pain^12^ and in opioid-induced hyperalgesia^13^ in healthy volunteers. In both trials pain ratings after CBD application did not significantly differ from placebo.

A placebo-controlled trial in hand osteoarthritis and psoriatic arthritis found no significant effect of CBD on pain intensity.^14^ However, the applied dosages were very low and far below those utilized in clinical trials of CBD in rare epilepsy syndromes, where its efficacy could be demonstrated.^15^, ^16^, ^17^, ^18^

A further randomized placebo-controlled trial on CBD in peripheral neuropathic pain treatment also reported no relevant reduction in pain levels.^19^ Again, the doses of CBD utilized were very far below those used in the trials in epilepsy.

The aim of our study was to investigate the effects of oral high-dose CBD administered over 8 weeks on pain, function and patient global assessment as an add-on to continued paracetamol in chronic symptomatic KOA.

## Methods

### Study design

This prospective, randomized, placebo-controlled, double-blind, parallel-group study was conducted at the Outpatient Clinic of the Department of Special Anaesthesia and Pain Therapy at the Medical University of Vienna. The protocol was approved by the local ethics committee (Ethics Committee of the Medical University Vienna, Borschkegasse 8b/E06, 1090 Vienna, Austria; Date of registration: 11/2019; Registration Number 2064; Investigator: Sibylle Pramhas, M.D.) and was registered at ClinicalTrials.gov (NCT04607603) by Sibylle Pramhas M.D., principal investigator. All patients gave written informed consent before beginning study procedures. Patients were recruited at the Outpatient Clinic of the Department of Special Anaesthesia and Pain Therapy at the Medical University of Vienna, by advertisement in news print and via social media platforms.

### Patients

Patients aged over 18–98 years with chronic knee pain were eligible. Patients were required to score ≥5 on the pain subscale of the Western Ontario and McMasters Universities Osteoarthritis Index (WOMAC). Patients also had to fulfill the ACR clinical criteria for KOA (knee pain and ≥4 criteria of the following: (i) age >50 years; (ii) morning stiffness of <30 min duration; (iii) crepitus on active motion; (iv) bony tenderness; (v) bony enlargement and (vi)no palpable warmth of the synovium).^20^ Additionally, radiographic or magnetic resonance imaging (MRI) confirmation of KOA was required. Participants had to be willing and able to give written informed consent and to comply to study requirements.

All medications or interventions for KOA pain must have been stable for at least two weeks prior to screening, and participants had to be willing to maintain a stable regimen throughout the study.

Exclusion criteria included: major depression present for over 12 months (defined as ≥18 points in the Beck's Depression Inventory); history of a psychoactive substance use disorder within the preceding 12 months; pregnancy; breast feeding; participation in a clinical trial 3 weeks preceding screening; allergy to study medication; severe coexisting diseases; impaired kidney function; impaired hepatic function; recent intra-articular corticosteroid or hyaluronic acid injection into the knee joint.

Opioids except for tramadol as rescue medication, benzodiazepines other than indicated at regular low doses for sleep disorders, NSAID, corticosteroids, and cannabinoid-based medications including recreational or medicinal cannabis were not allowed during the study period.

Women of child bearing age were required to use contraceptives during the study, and pregnancy tests were performed prior to the beginning and every month of the study period.

### Randomization and allocation concealment

Study medication for the entire study period was uniformly packaged and numbered by Hubertus Pharmacy (Spittal/Drau, Austria) that was not otherwise involved in the study. All study medication was delivered to Outpatient Clinic of the Department of Special Anaesthesia and Pain Therapy at the Medical University of Vienna prior to the initiation of the study in a single batch.

Participants were randomized to receive a medication number and the medication packages were allocated accordingly on-site. Randomization and allocation of medication number was performed by medical personnel not otherwise involved in any of the study procedures.

For placebo and verum batches the opaque capsules were identical.

Randomization was performed on a 1:1 basis (CBD:placebo), was computer-assisted (web-based software ‘randomizer’ (https://www.meduniwien.ac.at/randomizer/web/login.php)) and stratified by age (<40 years; 40–60 years; >60 years), sex and baseline (V1) WOMAC Pain Index (<7; ≥7).

All patients, investigators and study site personnel were blinded to group assignments throughout the study.

At follow-up, patients were asked which treatment group they believed to have belonged to, providing 3 possible standard answers: a) CBD, b) placebo, c) uncertain, to evaluate the success of patient blinding.

### Procedures

Hemp-derived CBD (purity >99,8%) was extracted by BioSynthesis Pharma Group (BSPG) Ltd., Sandwich, UK, then imported and formulated into capsules by the BSPG subsidiary Trigal Pharma GmbH, Austria. Capsules containing CBD (200 mg/capsule) and indistinguishable placebos were prepared in conformity with GMP. Identically produced CBD capsules from the same supplier have previously been used by independent authors in clinical trials on conditions other than KOA.^21^^,^^22^

During a two-week screening and wash-out period any medication specified in the exclusion criteria was discontinued and participants were started on a daily dosage of paracetamol 3 g which they were requested to maintain throughout the study for their chronic pain.

Patients were routinely tested for Δ^9^-tetrahydrocannabinol (THC) and opioid consumption once during screening.

Following the screening period, patients were randomized to fixed-dose CBD or placebo as add-on study medication.

By protocol, CBD was titrated in three equal steps within 7 days (first week) to a total of 600 mg/d, administered in three capsules, and maintained for the following 7 weeks. Participants had to take the trial medication t.i.d. with a meal for optimal CBD uptake.

If required, tramadol was allowed as rescue medication (up to 300 mg/d IR Tramal®, Grünenthal, Austria).

At the end of the 8-week maintenance phase, study medication was tapered off within one week.

On-site study visits were performed at screening (baseline), weeks 4, week 8 and at follow-up (week 12). In weekly telephone visits adverse events (AE) and pain level determined by numeric rating scale were recorded. Patients kept a paper diary of their daily consumption of tramadol rescue medication and their visual analogue scale (VAS) scores (10 cm VAS scale) in the morning and evening.

Venous blood tests (clinical chemistry, full blood count, coagulation) were performed and all outcome measures were evaluated at baseline (screening), week 4, week 8, and at follow-up (week 12).

A full medical history, physical examination, ECG, measurements of height, weight, and blood pressure were performed during screening and repeated at week 4, week 8 and follow-up.

There were no deviations from protocol.

### Outcome measures

Pain, stiffness and function were assessed utilizing the 11-point numeric rating scale WOMAC questionnaire.^23^^,^^24^

The primary outcome was ‘change in pain from baseline to week 8 of treatment’ by means of WOMAC pain subscale^25^^,^^26^ (0 = no pain, 10 = worst possible pain) during all evaluated activities.

Continuous secondary outcome parameters were also evaluated as ‘change from baseline to week 8 of treatment’ and were comprised of: WOMAC stiffness subscale (0 no stiffness, 10 worst stiffness); WOMAC physical function subscale (0 no functional impairment, 10 worst functional impairment); change in weekly mean baseline (screening) VAS-score to mean VAS-score during last week of the treatment phase (week 8) (0 no pain, 10 worst possible pain); PainDETECT score (range from 0 to 38^27^: score >19 = likelihood of neuropathic pain >90%; score 12–18 points = a neuropathic pain component may be present; score <12 points = neuropathic pain unlikely); SF-36 Questionnaire (zero = lowest, 100 = highest level of health)^28^; 6 min walk-test^29^; use of tramadol rescue medication; Patient Global Assessment of KOA (PGA-KOA) by asking the following standard question: ‘Considering all the ways your osteoarthritis of the knee affects you, how are you doing today on a scale from 1 = very good to 5 = poor’.

Response rates defined as a) ≥30% b) ≥50% reduction in WOMAC pain subscale and mean VAS-Score from baseline to the last week of treatment were compared between treatment groups.

Safety outcomes included frequency of adverse events per group, description of adverse events and monitoring of parameters of liver function.

A relevant elevation from baseline of the liver aminotransferases (ASAT/ALAT) and γ-glutamyltransferase (γ-GT) was defined as an (1) at least twofold increase from baseline at weeks 4 and 8, or (2) at least threefold rise from baseline at any time during the 8-week study period.

### Statistical analysis

A sample size of 43 patients per treatment group provides a power of 80% at a two-sided 5% significance level based on a minimal clinically relevant difference of 1 point on the WOMAC pain index scale between placebo and verum in the change of WOMAC pain index from baseline.

A standard deviation of 1.62 of the change in WOMAC pain index was assumed (see Table 2 in Conaghan et al.^24^).

Metric variables are described by medians and interquartile ranges or, where appropriate, by means and SDs. Frequencies are reported as counts and percentages.

The primary endpoint was analyzed using an analysis of covariance model with change from baseline to week 8 as dependent variable and randomization group, baseline score of WOMAC Pain and stratification variables as independent variables; least-squares means from this model and their group differences are reported with 95% confidence intervals (CI). Residual distributions were successfully checked for approximate normal distribution and potentially influential observations. (Quasi-)continuous secondary endpoints were analysed in the same manner.

Differences in intake of rescue medication between the randomization groups were compared by the Wilcoxon rank-sum test. Proportions of responders were compared between groups using a logistic regression model to adjust group comparison for stratification factors.

All analyses were based on the intention-to-treat principle, using multiple imputation to account for discontinuations (see Supplement).

Since a single primary endpoint at a single timepoint (8 weeks) had been pre-defined, no correction for multiple testing was performed. The results of the secondary endpoints are explicitly reported as *exploratory* results (without multiplicity correction) irrespective of their statistical significance.

Statistical analysis was performed using SAS version 9.4. Two-sided p-value of <0.05 was considered statistically significant.

### Role of funding source

This study was supported by Trigal Pharma GmbH, Vienna, Austria.

The funder of the study had no role in study design, data collection, data analysis, data interpretation or writing of the report.

## Results

Recruitment was conducted from October 1, 2020 to December 16, 2021, treatment was carried out from October 13, 2020 to February 17, 2022. The last follow-up visit was completed on March 29, 2022.

### Study population and participant flow

A total of 159 patients were screened by telephone and in person for eligibility, and 86 patients were randomized. Two patients discontinued prior to receiving the allocated intervention due to withdrawal of consent. After randomization, 42 patients received CBD and 42 patients received placebo. During study week 1 through week 4, six patients discontinued the study due to adverse events in the placebo arm and six in the CBD arm. In each arm one patient could no longer be contacted. During study weeks 5–8, four patients in the placebo arm discontinued. Of these, two patients had undergone an intervention not permitted in the protocol and two displayed insufficient compliance to study measures. In the CBD arm, seven patients discontinued during weeks 5–8 due to AE. One patient could no longer be contacted. There was no statistically significant difference in discontinuations between the CBD (n = 16) and the placebo arm (n = 12) (p = 0.48). Equally, there was no statistically significant difference in discontinuations due to AE between the CBD (n = 13) and the placebo arm (n = 6) (p = 0.12).

**Fig. 1:**
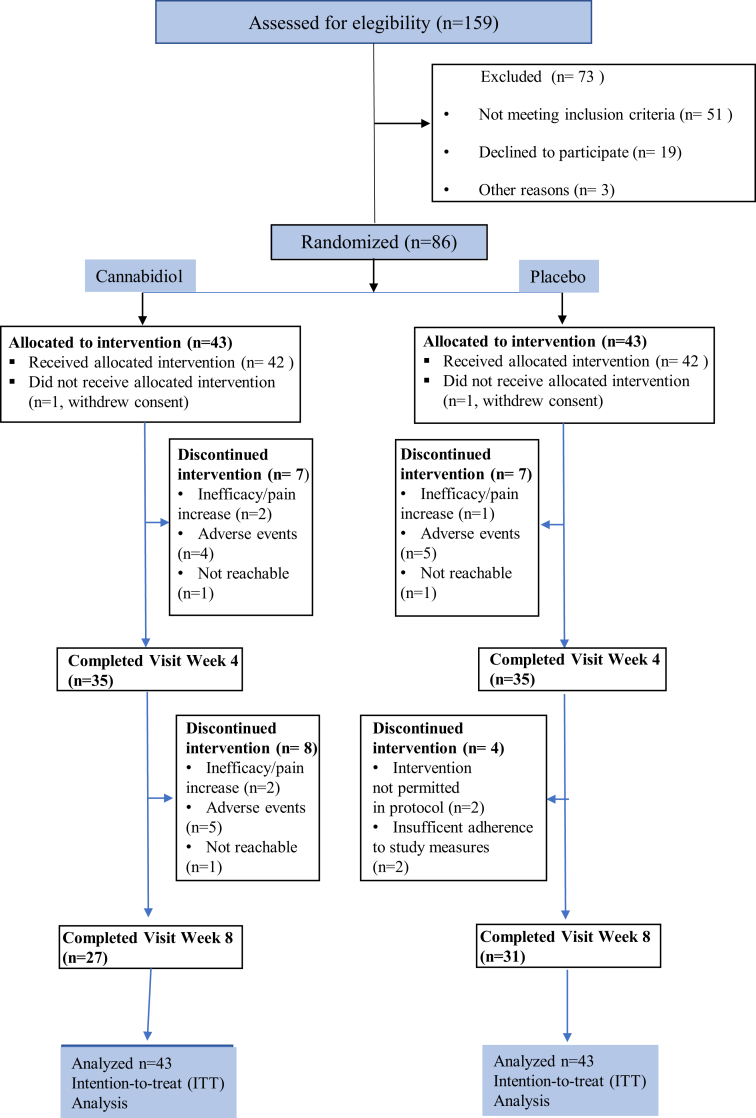
**CONSORT flow diagram of patient progress through the trial.** Patient progress through the trial including discontinuations.

Participant flow through the trial and discontinuations are depicted in Fig. 1.

Among the 86 participants, 60 were females [69.8%]. Patient demographic data and baseline characteristics are shown in Table 1.

**Table 1:** Baseline characteristics of the intention-to-treat population.

Variable	Treatment: cannabidiol (n = 43) female: n = 30; male: n = 13				Treatment: placebo (n = 43) female: n = 30; male: n = 13			
	Missing (n)	Median, lower and upper quartile	Minimum	Maximum	Missing (n)	Median, lower and upper quartile	Minimum	Maximum
Age, years	0	60.0 (55.0; 65.0)	38	92	0	65.0 (56.0; 73.0)	38.0	85.0
Body-mass-index (kg/m^2^)	0	28.0 (24.0; 31.4)	19.3	39.3	0	30.0 (26.0; 34.3)	21.4	42.7
WOMAC pain subscale	0	5.8 (5.0; 6.6)	4.6	10.0	0	5.6 (5.2; 6.4)	4.6	8.0
WOMAC stiffness subscale	0	7.0 (4.5; 8.0)	0	9.0	0	5.5 (3.5; 7.0)	0.0	10.0
WOMAC physical function subscale	0	5.3 (3.7; 6.4)	0.2	9.2	0	5.0 (4.3; 6.5)	1.5	8.9
Mean baseline (screening) VAS-score	0	5.7 (4.6; 6.4)	2.4	9.2	0	5.6 (4.7; 6.2)	2.5	8.7
PGA-KOA score	0	3.5 (3.0; 4.0)	2.0	5.0	0	4.0 (3.0; 4.0)	2.0	5.0
6-min walk-test,^29^ (m)	0	468.2 (413.1; 516.4)	0.0	688.5	1	426.3 (344.3–482.0)	186.0	619.7
PainDETECT score	0	15 (12.0; 21.0)	2.0	30.0	1	13.0 (7.0–17.0)	1.0	30.0
SF 36 physical component summary (PCS)	0	29.6 (26.1–37.2)	13.0	48.1	1	52.1 (45.2; 62.6)	29.6	68.1
SF 36 mental component summary (MCS)	0	55.1 (39.0; 61.7)	27.8	70.0	1	29.2 (23; 34.1)	13.9	47.3

WOMAC = Western Ontario and McMasters Universities Osteoarthritis Index; VAS = visual analogue scale; PGA-KOA = Patient Global Assessment of KOA; SF-36 = Short-Form-36 Health Survey.

Baseline grading of the severity of KOA by radiography (Kellgren–Lawrence classification) or MRI (chondropathy grade) are given in Table 2.

**Table 2:** Baseline grading of severity of osteoarthritis of the knee by radiography or magnetic resonance imaging.

	Treatment: cannabidiol (n = 43)	Treatment: placebo (n = 43)
Radiographic imaging: Kellgren–Lawrence classification		
Grade 2 (n)	7	10
Grade 3 (n)	5	12
Grade 4 (n)	8	9
Magnetic resonance imaging: chondropathy		
Grade 3 (n)	12	7
Grade 4 (n)	11	5

The observed baseline group differences in age, Body-Mass-Index, WOMAC stiffness subscale and 6-min walk-test were not judged clinically significant.

At baseline 24 patients (56%) in the CBD-group and 28 patients (65%) in the placebo group used no analgesic medication.

Oral NSAIDs were used by 14 patients (33%) CBD-group and 13 patients (30%) in the placebo group.

### Efficacy measures

#### Primary outcome: change in WOMAC pain subscale score

**Fig. 2:**
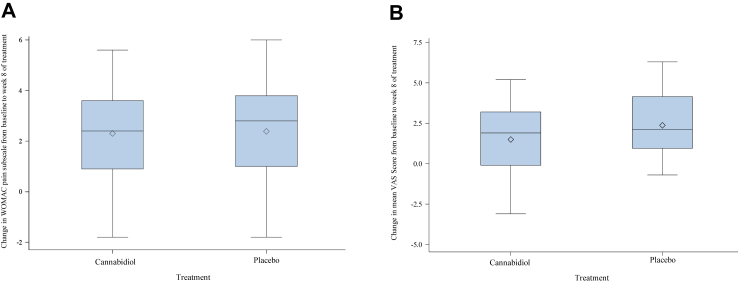
**Mean change in WOMAC pain subscale (A) and VAS-Score (B) from baseline to week 8 of treatment in the per protocol population.** (A) Per protocol population: Patients who completed entire study; Cannabidiol (n = 27), Placebo (n = 31), WOMAC = Western Ontario and McMasters Universities Osteoarthritis Index, WOMAC pain subscale ranging from 0 to 10 (higher scores indicate higher level of pain) x-axis: treatment group; y-axis Change in WOMAC pain subscale from baseline to week 8 of treatment (positive values indicate a reduction on the WOMAC pain subscale, negative values an increase). Whiskers: minimum and maximum values; Line: median; Diamond: mean; Lower end of box: lower quartile; Upper end of box: upper quartile. (B) Per protocol population: Patients who completed entire study; Cannabidiol (n = 27), Placebo (n = 31), VAS = Visual Analogue Scale ranging from 0 to 10 (higher scores indicate higher level of pain), recorded twice daily, mean of all days recorded during screening (baseline) and week 8 of treatment; x-axis: treatment group; y-axis: Change in mean VAS score from baseline to week 8 of treatment (positive values indicate a reduction in mean VAS Score, negative values an increase); Whiskers: minimum and maximum values; Line: median; Diamond: mean; Lower end of box: lower quartile; Upper end of box: upper quartile.

Mean score reductions in the primary outcome WOMAC pain subscale were 2.5 (95% CI: 1.8–3.3) in the CBD group and 2.4 (95% CI: 1.7–3.2) in the placebo group resulting in a mean group difference of 0.1 (95% CI: −0.8 to 1.0) (p = 0.80), which was not significant (Fig. 2A).

#### Secondary outcomes

Correspondingly, mean changes in all secondary endpoints from baseline to week 8 of treatment also did not significantly differ between the CBD and the placebo group. A synopsis of changes in continuous secondary outcomes is given in Table 3.

**Table 3:** Synopsis of changes in continuous secondary outcomes from baseline to week 8 of treatment.

Secondary outcome parameter	Mean change from baseline to last week of treatment: CBD group	Mean change from baseline to last week of treatment: Placebo group	Group difference CBD-Placebo	p-value
WOMAC physical function subscale	−1.5 (95% CI: 0.7–2.4)	−1.7 (95% CI: 0.8–2.5)	−0.1 (95% CI: −1.2 to 2.5)	0.81
WOMAC stiffness subscale	−2.4 (95% CI: 1.5–3.3)	−2.5 (95% CI: 1.5–3.4)	−0.1 (95% CI: −1.2 to 1.1)	0.90
PGA-KOA	−1.2 (95% CI: 0.8–1.7)	−1.0 (95% CI: 0.6–1.4)	0.2 (95% CI: −0.3 to 0.7)	0.40
Mean weekly VAS-score	−1.9 (95% CI: 1.1–2.7)	−2.4 (95% CI: 1.6–3.2)	−0.51 (95% CI: −1.5 to 0.5)	0.30
SF36 physical component summary (PCS)	+8.0 (95% CI: 4.3–11.5)	+7.3 (95% CI: 4.0–10.7)	0.65 (95% CI: −3.6 to 4.9)	0.80
SF36 mental component summary (MCS)	+0.34 (95% CI: −2.2 to 2.9)	−1.5 (95% CI: −4.0 to 1)	1.85 (95% CI: −1.2 to 4.9)	0.24
6 min walk-test	+8.0 m (95% CI: −27.8 to 43.2)	+29.0 m (95% CI: −10.1 to 67.7)	−21.1 (95% CI: −68.1 to 25.9)	0.38
PainDETECT score	−4.0 (95% CI: 1.8–6.3)	−3.0 (95% CI: 0.7–5.3)	1.0 (95% CI: −1.8 to 3.8)	0.47

Data are depicted as mean and 95% confidence interval (CI) estimated for the intention-to-treat analysis set (86 patients) by ANCOVA models.

WOMAC = Western Ontario and McMasters Universities Osteoarthritis Index; VAS = visual analogue scale; PGA-KOA = Patient Global Assessment of KOA; SF-36 = Short-Form-36 Health Survey.

Mean reduction in weekly VAS score was 1.9 (95% CI: 1.1–2.7) in the CBD group and 2.4 (95% CI: 1.6–3.2) in the placebo group (mean group difference −0.51 [95% CI: −1.5 to 0.5]) (p = 0.3) (Fig. 2B).

Similarly, response rates and frequency of tramadol consumption did not significantly differ between the two groups (Supplementary Table S1).

### Adverse events

Adverse events (AE) were extremely common in both study arms: 39 patients (93%) in the CBD arm experienced at least one AE, and 36 patients (88%) reported at least one AE in the placebo group (p = 0.48). Median number of AEs was 3 in CBD (quartiles: 2–5, range: 0–7) and 2 in placebo (quartiles: 2–4, range 0–6) (p = 0.13).

A total of 240 AE events were reported during the 8-week study period (Table 4).

**Table 4:** Frequency of adverse events in the cannabidiol group and placebo group.

Adverse event	Frequency CBD arm (n)	Frequency placebo arm (n)	p
Diarrhea/loose stools	19	20	n.s.
Elevation liver aminotransferases (ASAT/ALAT) or gamma-GT during active study period	15	5	0.022
Abdominal pain	14	11	n.s
Fatigue	14	10	n.s
Change in bowel habits	8	4	n.s
Vertigo	7	5	n.s
Gastroesophageal reflux	5	2	n.s
Nausea	4	8	n.s
Obstipation	4	2	n.s
Swelling knee/knee bursitis	3	1	n.s
Dry mouth	3	0	n.s
Pain in right upper quadrant	3	0	n.s
Changes in mood	3	0	n.s
Increased appetite	2	2	n.s
Loss of appetite	2	4	n.s
Flatulence	2	3	n.s
Weight loss	2	0	n.s
Sleep disturbance	2	1	n.s
Dysgeusia	2	0	n.s
Increased sweating	2	1	n.s
Tachycardia/palpitations	2	0	n.s
Hair loss	1	0	n.s
Weight gain	1	1	n.s
Blurred vision	1	0	n.s
Fall/joint contusion	1	1	n.s
Swallowing complaints	1	0	n.s
COVID-19 infection	1	0	n.s
Impaired concentration	1	1	n.s
Hypotension	1	1	n.s
Headache	1	6	n.s
Hematochezia	1	0	n.s.
Urinary tract infection	1	0	n.s
Vivid dreams	1	0	n.s
Conjunctivitis	1	0	n.s
Flank pain	1	1	n.s
Respiratory tract infection	1	4	n.s
Changes in urine colour	1	0	n.s
Epistaxis	1	0	n.s
Edema of the eyelids	0	1	n.s
Toothache	0	1	n.s
Joint stiffness	0	1	n.s
Elevation of creatinine	0	1	n.s
Shivering	0	1	n.s
Erythema	0	1	n.s
Erysipelas	0	1	n.s
Hot flushes	0	1	n.s
Drowsiness	0	2	n.s
Pruritus	0	1	n.s

135 AE occurred in the CBD group (56%) and 105 AE in the placebo group (44%), resulting in a significantly larger cumulative number of AE (p = 0.008) in the CBD group.

Diarrhea was the most common AE in both groups, followed by abdominal pain and fatigue, whereas serum liver aminotransferase and γ-glutamyltransferase (γ-GT) elevations were predominantly seen in CBD-treated patients (Table 4).

Rise above baseline of liver aminotransferases (ASAT/ALAT) and γ-GT was significantly more common in the CBD group (n = 15) than in the placebo group (n = 5) (p = 0.02), but many of these elevations were mild and clinically irrelevant.

Occurrence of relevant ASAT and ALAT elevations fulfilling the predefined criteria was not significantly more frequent in the CBD group: ASAT elevation (CBD: n = 2; placebo n = 0; [p = 0.494]); ALAT elevation (CBD: n = 3 placebo n = 0; [p = 0.24]).

Relevant γ-GT elevations meeting the predefined criteria were significantly more frequent in the CBD group (n = 11) than in the placebo group (n = 0) (p < 0.001).

At follow-up visit 4 weeks after cessation of study medication, all elevations described above for ASAT, ALAT and γ-GT had fully resolved.

The course of relevant ASAT, ALAT and γ-GT elevations is given in Supplementary Tables S3–S5 respectively.

### Paracetamol dosage

A total of 24 patients (27.9%) slightly reduced their paracetamol maintenance dose during the course of the study (12 patients from the placebo group and 12 patients from the CBD group).

At study week 4, a paracetamol dosage of 3 g/d was maintained by 71.4% of still participating patients in the CBD as well as the placebo group. At study week 8, 63.0% of still participating patients were on a paracetamol dosage of 3 g/d in the CBD group and 64.5% in the placebo group.

The odds ratio for a paracetamol dosage of 3 g/d at study week 8 was 0.76 (95% CI: 0.27–2.17) in the CBD as compared to the placebo group (p = 0.61).

### Blinding efficacy

At follow-up, 77 patients were available for survey of blinding efficacy. In 37 patients (48%) the answer to the survey question corresponded to the actual treatment allocation (Supplementary Table S2), demonstrating even odds and thus effective blinding throughout the study.

## Discussion

In this trial, plant-derived high-dose oral CBD given for 8 weeks as an add-on to continued paracetamol was not superior to continued paracetamol alone for the treatment of pain or impaired function in KOA patients. The primary outcome (change in WOMAC pain subscale over the 8-week study period) did not significantly differ between the placebo and the CBD group. This was also true for all secondary outcome parameters measured.

In preclinical studies, CBD has shown promise as an anti-inflammatory drug in murine models of acute inflammation and osteoarthritis^4^ and in spontaneous OA in dogs.^5^^,^^6^

In contrast to these animal data, but in congruence with our results, previous clinical trials did not find a significant analgesic effect of CBD. One trial evaluated CBD in hand and psoriatic arthritis.^14^ Another randomized clinical trial in peripheral neuropathic pain also reported no relevant analgesic effect of CBD.^19^ The results of these trials, however, were difficult to interpret due to the very small daily doses of CBD used (20–50 mg/d), which could be ineffective due to underdosing. A further trial in acute postoperative pain^11^ only showed a significant analgesic effect of CBD on the first postoperative day, which was lost on all subsequent days. Again, the doses used (75 and 150 mg/d) were small. A further negative trial in acute low back pain only applied a single dose of 400 mg oral cannabidiol.

As far as we are aware, our present paper reports the first randomized, placebo-controlled clinical trial on the potential analgesic efficacy of oral CBD in osteoarthritis using high dosages and a treatment duration very close or identical to those employed in published clinical trials of its antiepileptic efficacy.^15^, ^16^, ^17^^,^^30^

Considering our predominantly elderly study population, the overall tolerability of CBD 600 mg/d as an add-on to continued paracetamol was good, with no serious AE observed in this relatively small sample of KOA patients.

Although relevant elevations of ASAT, ALAT and γ-GT were significantly more often detected in the CBD group, all elevations fulfilling the relevance criteria spontaneously returned within the normal range or baseline during the follow-up period of 4 weeks after cessation of CBD. Our findings correspond well to those reported from previous clinical trials^15^, ^16^, ^17^ in young epilepsy patients. Close monitoring of liver parameters is therefore recommended and should be mandatory when commencing CBD therapy.

In this trial the fixed dose of 600 mg/d oral CBD (resulting range of individual dose: 4.6–10 mg × kg^−1^ × d^−1^) was much higher than in any previous CBD trial for chronic pain and was close to those in previous clinical trials on epilepsy (5–20 mg × kg^−1^ × d^−1^).^15^, ^16^, ^17^^,^^30^ Nevertheless, we cannot exclude that efficacy may have been limited by relative underdosing.

As CBD plasma levels were not determined, variable resorption also cannot be excluded. However, across a former independently published trial with identically produced CBD capsules from the same source of supply given twice daily,^21^ the achieved plasma CBD concentrations in adults were shown to be stable and within the theoretically expected range.

Though patients were encouraged to maintain a stable paracetamol dosage, 28% occasionally reduced dosage during the study. Reductions were equally distributed between CBD and placebo group, but this circumstance could theoretically complicate a straightforward interpretation. There is general consensus on a rather low or almost negligible analgesic potency of paracetamol in low back pain and painful OA.^2^^,^^3^^,^^31^^,^^32^ Although the study design cannot absolutely rule out an ability of paracetamol to partially hide a minor analgesic effect of CBD in painful KOA, our trial did not point to such an analgesic activity of CBD. The clinical relevance of a potential analgesic contribution of CBD, small enough to escape our present investigational approach, should be considered more than questionable.

In conclusion, our results do not support the yet clinically unproven hopes for CBD as potential supplement or even replacement of potent analgesics, including opioids.^7^

## Contributors

S.P. and H.G.K. designed the study and wrote the study protocol. S.P. and H.G.K. managed the literature searches and analyses. S.P., T.T., S.T., D.P., and S.S. contributed to the data collection. S.P. and H.G.K. wrote the first draft of the manuscript and critically revised all subsequent versions for important intellectual content. A.G., S.S., T.T., S.T., and D.P. contributed to the subsequent drafting of the manuscript. S.S. and H.G.K. provided the infrastructure for the data collection. A.G. and S.P. undertook the statistical analysis. All authors had full access to all the data in the study.

All authors have given final approval of the version to be published and agreed to be accountable for all aspects of the work in ensuring that questions related to the accuracy or integrity of any part of the work are appropriately investigated and resolved.

## Data sharing statement

De-identified patient data and data dictionary that underlie the results reported in this manuscript can be made available to investigators for research purposes on a case-by-case basis with publication and after approval of research proposal by all co-authors. A signed data access agreement will be required. Requests for access to data should be addressed to the corresponding author at sibylle.pramhas@meduniwien.ac.at.

## Declaration of interests

We declare no competing interests.

## Appendix A Supplementary data

Supplementary Material

Efficacy_CBD_Knee_OA_10_09_2021_Version_1_5
