# Supplementary material for: Oral cannabidiol (CBD) as add-on to paracetamol for painful chronic osteoarthritis of the knee: a randomized, double-blind, placebo-controlled clinical trial
Source: Lancet Reg Health Eur. 2023 Nov 10;35:100777. doi: 10.1016/j.lanepe.2023.100777 (PMC10682664; doi:10.1016/j.lanepe.2023.100777)
Supplement: Supplementary Material [file mmc1.docx]

| Secondary Outcome parameter (categorical) | Frequency CBD Group  (n) | Frequency Placebo Group  (n) | p-value |
| --- | --- | --- | --- |
| Response: ≥30% reduction in mean VAS-Score from baseline to the last week of treatment^1^ | 14 | 18 | p=0.66 |
| Response: ≥50% reduction in mean VAS-Score from baseline to the last week of treatment^2^ | 8 | 13 | p=0.30 |
| Response: ≥30% reduction in WOMAC pain subscale from baseline to the last week of treatment^3^ | 16 | 23 | p=0.364 |
| Response: ≥50% reduction in WOMAC pain subscale from baseline to the last week of treatment^4^ | 11 | 15 | P=0.945 |
| Use of IR tramadol ^5^ | 3 | 4 | p=0.71 |

**Supplementary Table 1: Repones Rates and Use of Rescue Medication**

.

^1^ Response defined as: ≥30% reduction in mean VAS-Score from baseline to the last week of treatment, odds for response were 22% lower in the CBD group than in the placebo group (Odds ratio (OR) = 0.78, 95%CI: 0.26 to 2.33; p=0.66).

^2^Response defined as: ≥50% reduction in mean VAS-Score from baseline to the last week of treatment, odds for response were 47% lower in the CBD group than in the placebo (Odds ratio (OR) = 0.53, 95%CI: 0.16 to 1.75; p=0.30)

^3^ Response defined as: ≥30% reduction in WOMAC pain subscale from baseline to the last week of treatment, odds for response were 38% lower in the CBD group than in the placebo group (Odds ratio (OR) = 0.62, 95%CI: 0.22 to 1.73; p=0.66).

^4^ Response defined as: ≥50% reduction in WOMAC pain subscale from baseline to the last week of treatment, odds for response were 3% lower in the CBD group than in the placebo group (Odds ratio (OR) = 0.97, 95%CI: 0.35 to 2.64; p=0.945).

^5^Fisher's Exact Test p=0.71

**Supplementary Table 2. Blinding Survey**

|  | **Survey: CBD** | **Survey: Placebo** | **Survey: Uncertain** |
| --- | --- | --- | --- |
| **Allocation** |  |  |  |
| CBD (n=40) | 17 (42.5%) | 9 (22.5%) | 14 (35%) |
| Placebo (n=37) | 9 (24%) | 20 (54%) | 8 (22%) |

**Supplementary Table 3. Course of relevant ASAT elevations (U/l) (n=2)**

| **Patient Number** | **Treatment** | **ASAT (U/l)** | | | |
| --- | --- | --- | --- | --- | --- |
|  |  | **Baseline** | **Week 4** | **Week 8** | **Follow -Up** |
| **23** | **CBD** | **20** | **134** | **-** | **17** |
| **50** | **CBD** | **20** | **18** | **78** | **19** |

**Supplementary Table 4. Course of relevant ALAT elevations (U/l) (n=3)**

| **Patient Number** | **Treatment** | **ALAT (U/l)** | | | |
| --- | --- | --- | --- | --- | --- |
|  |  | **Baseline** | **Week 4** | **Week 8** | **Follow -Up** |
| **23** | **CBD** | **27** | **163** | **-** | **21** |
| **50** | **CBD** | **13** | **21** | **250** | **20** |
| **53** | **CBD** | **24** | **65** | **60** | **48** |

**Supplementary Table 5. Course of relevant γ-glutamyltransferase (****γ-GT) elevations (U/l) (n=11)**

| **Patient Number** | **Treatment** | **γ-glutamyltransferase (γ-GT) elevations (U/l)** | | | |
| --- | --- | --- | --- | --- | --- |
|  |  | **Baseline** | **Week 4** | **Week 8** | **Follow -Up** |
| 23 | CBD | 15 | 90 | - | 28 |
| 33 | CBD | 32 | 76 | 101 | 53 |
| 38 | CBD | 25 | 69 | 67 | 29 |
| 45 | CBD | 36 | 106 | 102 | 46 |
| 50 | CBD | 7 | 8 | 86 | 23 |
| 53 | CBD | 15 | 66 | 145 | 60 |
| 55 | CBD | 68 | 198 | 333 | 73 |
| 61 | CBD | 16 | 61 | 25 | 19 |
| 69 | CBD | 25 | 36 | 125 | 37 |
| 83 | CBD | 47 | 240 | . | 58 |
| 84 | CBD | 16 | 92 | . | 42 |

**Further details on statistical analyses**

For each outcome the following imputation approach was used: multiple imputation was applied separately in each randomization group, assuming missingness at random (MAR). Missing outcome values were first imputed by producing a monotone missingness pattern using the MCMC method with 33 imputations (corresponding to 33% of missing data). Next, regression models with predictive mean matching were applied to each imputed data set from the ﬁrst step. Both imputation steps used all existing outcome values from visits 1 to 4 as well as age, sex and the WOMAC pain baseline score as covariables in the imputation models. The ﬁnal results were obtained by applying Rubin’s rules to the results from the imputed data sets (proc mianalyze in SAS).
