# Supplementary material for: Oral cannabidiol (CBD) as add-on to paracetamol for painful chronic osteoarthritis of the knee: a randomized, double-blind, placebo-controlled clinical trial
Source: Lancet Reg Health Eur. 2023 Nov 10;35:100777. doi: 10.1016/j.lanepe.2023.100777 (PMC10682664; doi:10.1016/j.lanepe.2023.100777)
Supplement: Efficacy_CBD_Knee_OA_10_09_2021_Version_1_5 [file mmc2.pdf]

Efficacy of Cannabidiol in Treatment of Pain due to symptomatic Osteoarthritis of the Knee: A randomized, double-blind, placebo-controlled Clinical Trial

Clinical Investigator:

Dr. Sibylle Pramhas

Department of Anesthesiology and Pain Medicine, Medical University Vienna

Währingergürtel 18-20

A-1090, Vienna

Protocol Authors

Dr. Sibylle Pramhas, O.Univ.Prof. DDr. Hans-Georg Kress, FFPMCAI

Sponsor:

Department of Anesthesiology and Pain Medicine, Medical University Vienna

Währingergürtel 18-20

A-1090, Vienna

Trial centre:

Department of Anesthesiology and Pain Medicine

Währingergürtel 18-20

A-1090, Vienna

EudraCT-Nr: 2019-003591-40

SPONSOR, INVESTIGATOR, AND SIGNATURES

Sponsor/or representative (AMG §§ 2a, 31, 32)

Medical University of Vienna, Austria

Signature	Date

Investigator (AMG §§ 2a, 35, 36)

Dr. Sibylle Pramhas, Department of Anesthesiology and Pain Medicine, Medical University of Vienna, Austria

Signature	Date

List of Abbreviations

OA	Osteoarthritis
NSAID	Non-steroidal anti-inflammatory drugs
COX	Cyclooxygenase
CV	cardiovascular
GI	gastrointestinal
CBD	Cannabidiol
THC	Δ^9-tetrahydrocannabinol (THC)
PGA-KOA	patient global assessment of knee osteoarthritis
WOMAC	Western Ontario and McMasters Universities Osteoarthritis Index
VAS	Visual Analogue Scale
BDI	Beck Depression Inventory

Efficacy of Cannabidiol in Treatment of Pain due to symptomatic Osteoarthritis of the Knee: A randomized, double-blind, placebo-controlled Clinical Trial

Introduction

Osteoarthritis (OA) of the knee is a very common condition. Symptomatic OA of the knee has a prevalence of 9.5% (age 63-94 years) and the incidence of knee OA in adults aged 20-84 years is 240/100,000. Accordingly, symptomatic knee OA accounts for a significant proportion of disability and pain in middle-aged and older adults, worldwide [1].

Systemic pharmacological treatment options in painful symptomatic knee OA are limited. The current Osteoarthritis Research Society International (OARSI) guidelines recommend paracetamol, duloxetine, oral non-selective NSAIDs and oral COX-2 inhibitors as appropriate in individuals *without relevant comorbidities*.

In individuals *with* relevant co-morbidities (diabetes; advanced age; hypertension; CV disease; renal failure; GI complications; depression; physical impairment limiting activity, including obesity) the recommendation changes to *uncertain* for paracetamol, oral non-selective NSAIDs and oral COX-2 inhibitors.

In individuals with high co-morbidity risk (history of GI-bleeding, myocardial infarction, chronic renal failure) NSAIDs and oral COX-2 inhibitors are evaluated as inappropriate.

The recommendation for opioids, be it in transdermal or oral application, is uncertain in all patient groups due to an unfavorable risk-benefit ratio.

In all of the above drugs the OARSI guideline call for conservative dosing and treatment duration.

Many patients presenting with knee osteoarthritis are of an advanced age and suffer from various co-morbidities. The benefit of the available systemic pharmacological treatment options in these

patients can be summarized as uncertain. Therefore, the investigation of new symptomatic systemic pharmacological treatment options for knee OA is relevant.

Cannabidiol (CBD) one of the major alkaloid components isolated from cannabis. In contrast to Δ^9-tetrahydrocannabinol (THC) it does not display hallucinogenic activity and is not a scheduled narcotic drug.

CBD exhibits neuroprotective, antiepileptic, anxiolytic, antipsychotic and anti-inflammatory properties. [2]

Its safety and efficacy in the treatment of two rare forms of epilepsy, Lennox-Gastaut Syndrome and Dravets Syndrome has been demonstrated [3-5]

CBD displayed anti-inflammatory properties in various preclinical investigations in animal models.

In a mouse model of acute lung injury a single dose of cannabidiol (20 mg/kg) administered prior to the induction of LPS (lipopolysaccharide)-induced acute lung injury decreases leukocyte (specifically neutrophil) migration into the lungs, albumin concentration in the bronchoalveolar lavage fluid, myeloperoxidase activity in the lung tissue, and production of pro-inflammatory cytokines (TNF and IL-6) and chemokines (MCP-1 and MIP-2) 1, 2, and 4 days after the induction of LPS-induced acute lung injury[6]

In cerulein-induced acute pancreatitis in mice intraperitoneal cannabidiol treatment significantly improved the pathological changes and decreased the enzyme activities, IL-6 and tumor necrosis factor α levels, and the myeloperoxidase activities in plasma and in the organ tissues[7].

In a study by Philpott et al. osteoarthritis was induced in rats by intra-articular injection of sodium monoiodoacetate. Intraarticular injection of CBD in these animals decreased joint afferent firing rate, and attenuated pain behavior in a dose dependent manner [8].

Anti-arthritic effects of cannabidiol were also shown in a murine model of collagen-induced arthritis [9].

In a rat model of acute inflammation after intraplantar injection of carrageen anti-inflammatory and anti-hyperalgesic effects mediated by CBD binding of α3 glycine receptors were reported [10]

Another study in rats showed that daily oral treatment with cannabidiol reduced hyperalgesia to thermal and mechanical stimuli after sciatic nerve chronic constriction (neuropathic pain model) and complete Freund's adjuvant intraplantar injection (inflammatory pain model). Cannabidiol's activity was associated with a reduction in the content of several mediators, such as prostaglandin E2 (PGE2), lipid peroxide and nitric oxide (NO), and in the over-activity of glutathione-related enzymes [11].

A recent review of the efficacy of cannabis-based medicines for pain management concluded that they might be effective for the treatment of chronic pain [12].

However, the potential role of cannabidiol is unclear as none of the included studies investigated cannabidiol without the addition of THC. Furthermore, the doses of cannabidiol applied were far below those utilized in the trials investigating its efficacy in epilepsy.

The aim of the proposed study is to investigate the effects of cannabidiol in treatment of pain, function and patient global assessment due to symptomatic knee osteoarthritis.

Primary Objective/Hypothesis

To evaluate the efficacy of cannabidiol in reducing pain due to knee osteoarthritis, as compared to placebo.

Efficacy will be evaluated using the Western Ontario and McMasters Universities Osteoarthritis Index (WOMAC) Pain score.

Secondary Objectives

To assess the following in patients with painful knee osteoarthritis taking cannabidiol:

-Change of function in knee osteoarthritis

-Change of patient global assessment of knee osteoarthritis (PGA-KOA).

-Change of pain from baseline as compared to placebo measured by the Visual Analogue Scale (VAS)

-Responder rate in terms of change of pain from baseline

-Safety and efficacy of cannabidiol through monitoring of:

Adverse events

Clinical laboratory tests

Vital signs

-Health Related Quality of Life Score (SF-36) as compared to placebo

-6 min walk-test as compared to placebo

-PainDETECT score as compared to placebo

-to compare the use of the rescue medication in the placebo and treatment arm.

Primary Endpoints:

- Change from baseline (V1 see Table 1) in WOMAC-Pain Index to the last week of the treatment phase (V9, see Table 1)

Secondary Endpoints

- Change from baseline in WOMAC Physical function (V1) during the last week of the treatment phase (V9)

- Change from baseline (V1) in Patient global assessment of Knee Osteoarthritis (PGA-KOA) to PGA-KOA during the last week of the treatment phase (V9).
- Use of rescue medication
- Change from baseline (week 1) VAS-score during the last week of the treatment phase (week 9)
- Weekly mean of visual analogue scale (VAS)-score in the last week of the treatment period
- Number of patients considered treatment responders defined as
  those with a ≥30% reduction in mean VAS-Score from baseline to the last week of treatment
- Number of patients considered treatment responders defined as
  those with a ≥50% reduction in mean VAS-Score from baseline to the last week of treatment
- Safety assessment (Frequency of AE; changes in laboratory parameters; vital signs)
- Changes from baseline in quality of life as assessed by the SF-36 Questionaire
- Changes from baseline in 6 min walk-test
- Changes from baseline in PainDETECT score

Study Rationale

Many patients presenting with knee osteoarthritis are of an advanced age and suffer from various co-morbidities. The benefit of the available systemic pharmacological treatment options in these patients can be summarized as uncertain. Therefore, the investigation of new symptomatic systemic pharmacological treatment options for knee OA is relevant. If we are able to show a benefit for patients in the cannabidiol arm, the substance may constitute a further treatment alternative in knee osteoarthritis

Cannabidiol was well tolerated in prior clinical trials (see also safety assessment). We therefore consider that the risk-benefit assessment of the current study is favorable.

Study Design:

Prospective, randomized, double-blind placebo-controlled parallel group study (1:1 randomized).

Study population: Patients suffering from painful symptomatic knee osteoarthritis under stable treatment with paracetamol (3x 1g PD).

After a 2-week screening and wash-out period eligible patients will be included in the study. All patients will be required to be on paracetamol 3 x 1g PD as their basic analgesic medication.

Patients will be required to complete the Western Ontario and McMasters Universities Osteoarthritis Index (WOMAC) Pain, WOMAC Physical Function and PGA-KOA at V1, V6 and V9 and follow-up. (see also Table 1.)

PGA-KOA will consist of the following question: 'Considering all the ways your osteoarthritis of the knee affects you, how are you doing today on a scale from 1=very good to poor=5.

Patients will also be asked to keep a paper pain diary (VAS-Score twice daily). Patients will be required to discontinue all analgesic medication except for that specified in the protocol.

Patients will be allowed tramadol drops (50mg up to 6 times per day) as rescue medication.

During wash-out and screening patients will be screened for Δ^9-tetrahydrocannabinol (THC) and opioid consumption.

86 patients will be randomized to receive either 600mg cannabidiol/d or placebo on a 1:1 basis (43 patients per treatment group). Randomization will be computer-assisted (web-based software 'randomizer' (https://www.meduniwien.ac.at/randomizer/web/login.php)) and stratified by age (<40 years; 40-60 years; >60 years) and sex and baseline (V1) WOMAC Pain Index (<7; >7).

Allocation concealment is guaranteed by restricting handling and packaging of study drug to medical personnel *not* involved in any of the study procedures.

Randomization will be carried out by a web-based software ('randomizer,).

Upon randomization, each patient will be allocated a medication number. (see also Section: Randomization, Stratification and Blinding).

The blinding table will only be unblinded at time of analysis.

The treatment period will be 8 weeks long and will consist of a 6 days titration phase followed by a 7-week maintenance phase. In the verum group patients will be titrated to an oral dosage of 600mg cannabidiol per day administered in two equal doses (see dosage titration scheme).

Handling of study drug will be carried out by medical personnel not involved in study procedures.

The treatment period will be followed by a 6-day taper. During treatment and taper period weekly visits will be performed. Visits V1, V6, V9, V11 (see table below) will be performed at our out-patient clinic. The remaining visits will be performed via telephone.

A final follow-up visit (V11) will be carried out 2 weeks after the taper period. At follow-up the patients will be questioned as to whether they think they participated in the verum or placebo group.

The total study duration will be 13 weeks including screening and follow-up.

Anticipated duration first patient in (FPI) to last patient out (LPO) is 24 months.

For procedures and questionnaires performed at each visit please see Table 1.

Inclusion Criteria

- Patient must be willing and able to give informed consent for participation in the study
- Age 18-98 years
- Knee Pain
- WOMAC Pain Subscale ≥ 5 during screening
- Fulfilment of the clinical criteria of the American College of Rheumatology for knee OA [13]
- X-ray or MRI confirmation of knee osteoarthritis within the previous 12 months
- All medications or interventions for pain due to knee osteoarthritis

must have been stable for two weeks prior to screening and patient is willing to maintain a stable regimen throughout the study.

Exclusion Criteria:

- Current mood disorder (dysthymia, bipolar mood disorder)
- Major Depression > 12 months (Beck Depression Inventory Score ≥ 18)
- History of a psychoactive substance use disorder within the preceding 12 months
- Major coexisting medical illness (e.g. severe heart failure, pulmonary hypertension, renal insufficiency)
- Glaucoma
- Acute myocardial infarction
- Uncontrolled hypertension
- History of convulsion
- Pregnancy; women of childbearing age will be required to use contraceptives during the duration of the study. Furthermore, a pregnancy test will be performed prior to the beginning of the study and once a month during the study period.
- Breast feeding
- Participation in a clinical trial in the 3 weeks preceding the study
- Allergy to study medication
- Recent intra-articular corticosteroid or hyaluronic acid injection in the knee joint. Patients must be willing to abstain from such interventions during the entire study
- Use of the following medication:
    - opioids except for tramadol,
    - benzodiazepines other than indicated at low doses for sleep disorders
    - **NSAID**

  - Corticosteroids

- Impaired kidney function (Creatinine > 1.5mg/dl)
- Patient has significantly impaired hepatic function

  defined as **any** of the following:

  − Alanine aminotransferase (ALT) or aspartate aminotransferase

  (AST) >5 × upper limit of normal (ULN).

  − ALT or AST >3 × ULN **and** (total bilirubin [TBL] >2 × ULN

  or international normalized ratio [INR] >1.5).

  − ALT or AST >3 × ULN with the presence of fatigue, nausea, vomiting, right upper

  quadrant pain or tenderness, fever, rash, and/or eosinophilia (>5%).
- Patient is currently using or has in the past used recreational or

  medicinal cannabis or synthetic cannabinoid based medications within 3 months prior to study entry
- Patient is unwilling to abstain from using recreational or medicinal

  cannabis, or synthetic cannabinoid based medications during the study
- Patients who are not able to understand the study measures and are not able to complete pain assessment forms.

Sample Size Calculation

A sample size of 43 patients per treatment group was determined to provide a power of 80% at a two-sided significance level of 5% based on a minimal clinically relevant difference of 1 point on the WOMAC Pain Index scale between placebo and verum in the change in WOMAC Pain Index from baseline.

The estimate was based on a previous study by Conaghan et al. [14] where placebo-response was reported as a change from baseline of 1.4 in WOMAC Pain Index[9] . A standard deviation of 1.62 of the change in WOMAC Pain Index was assumed (see Table 2 in Conaghan et al).

Statistics

Metric variables will be described by medians and interquartile ranges or, where appropriate, by means and SDs. Frequencies will be reported as counts and percentages.

The primary end-point will be analyzed using an analysis of covariance model with terms for randomization group, baseline score of WOMAC-Pain and stratification variables. (Quasi-)continuous secondary endpoints will be analysed in the same manner.

Differences in intake of rescue medication between the randomization groups will be compared between groups by the Wilcoxon rank-sum test. Proportions of responders will be compared between groups using a Chi-square test.

All analyses will be based on the intention-to-treat principle, using multiple imputation to account for drop-outs.

Since a single primary endpoint has been defined (where also a single timepoint of interest has been selected) no correction for multiple testing will be performed. The results of the diverse secondary endpoints will be explicitly reported as *exploratory* results (without multiplicity correction) irrespective of their statistical significance.

Statistical analyses will be performed using SAS version 9.4. A two-sided p-value of <0,05 will be considered statistically significant.

Table 1. Study Design/Visits

Evaluation	Screening/<br>Wash-out	Titration	Maintenance Period							Taper<br>Period	Follow-up
Visit	V1	V2	V3	V4	V5	V6	V7	V8	V9	V10	V11
Week	W1-2	W3	W4	W5	W6	W7	W8	W9	W10	W11	W13
Informed Consent	X										
Exclusion/Inclusio<br>Criteria	x										
Randomization		x									
painDETECT	x					x			x		
Beck Depression Inventory	x								x		
Quality of Life<br>Score<br>SF-36	x								x		
WOMAC	x					x			x		x
PGA-KOA											x
6 minutes walking test	x					x			x		x
Mean weekly<br>VAS-Score	x	x	x	x	x	x	x	x	x	x	x
ECG	x					x			x		x
Physical<br>Examination	x					x			x		x
Height and Weight	x					x			x		x
Blood Pressure	x					x			x		x
Safety<br>Laboratory	x					x			x		x
Dosage<br>Titration		x									
Urine THC and opioid<br>screen	x										
Medical<br>History	x										
Diagnostic Imaging of the<br>Knee (MRI or X-Ray)	x										
Side Effects/<br>Adverse Events		x	x	x	x	x	x	x	x	x	x
Blinding<br>Survey											x
Pregnancy Test	x			x			x				x

Randomization, Stratification and Blinding

Study medication for the entire study period will be uniformly packaged and numbered by Hubertus Pharmacy (Spittal/Drau, Austria) which is not otherwise involved in the study procedures.

The manufacturer will supply CBD dosage 200mg and placebo in identical opaque capsules. After inclusion patients will be randomized to receive either 600mg cannabidiol/d or placebo on a 1:1 basis (43 patients per treatment group). Randomization will be computer-assisted (web-based software 'randomizer' (https://www.meduniwien.ac.at/randomizer/web/login.php)) and stratified by age (<40 years; 40-60 years; >60 years) and sex and baseline WOMAC Pain Index (<7; >7).

Randomization will be carried out by a member of staff not involved in any of the study procedures.

In case of drop-out medication will be held at our clinic until completion of the entire study (i.e. until unblinding and data analysis have been carried out).

Unreturned medication will be protocolled in the paper CRF.

Patients will be numbered in order of inclusion and randomization and receive a medication number according to randomization.

The blinding table will be available to the member of staff responsible for randomization online.

Unblinding will only be carried out at time of analysis.

If emergency unblinding is necessary the blinding table will be available at all times through the physician on duty at our clinic.

Intervention

After screening, inclusion, informed consent and randomization patients in the cannabidiol group will be titrated to a total dosage of 600mg CBD per day (see dosage titration scheme), administered in three daily doses, per os.

Table 2. Dosage titration Scheme

	**DAY 1**	**DAY 2**	**DAY 3**	**DAY 4**	**DAY 5**	**DAY 6**	**DAY 7**
**Cannabidiol Dosage, p.o.**	Placebo- -Placebo- 200mg	Placebo- -Placebo- 200mg	Placebo- -Placebo- 200mg	200mg- -Placebo- 200mg	200mg -Placebo- 200mg	200mg -Placebo- 200mg	200mg -200mg- 200 mg

Table 3. Dosage taper Scheme

	**DAY 1**	**DAY 2**	**DAY 3**	**DAY 4**	**DAY 5**	**DAY 6**	**DAY 7**
**Cannabidiol Dosage, p.o.**	200mg CBD- -Placebo- 200mg CBD	200mg CBD- -Placebo- 200mg CBD	200mg CBD- -Placebo- 200mg CBD	Placebo- -Placebo- 200mg CBD	Placebo -Placebo- 200mg CBD	Placebo -Placebo- 200mg CBD	Placebo -Placebo- Placebo

Placebo will also be administered in three doses at equal volumes without active ingredient per os.

Clinical laboratory samples (urine and blood) will be taken for hematology, biochemistry, coagulation, a urine tetrahydrocannabinol (THC) screen and a serum pregnancy test (if appropriate) at V1 (see Table 1).

A urine THC test will be performed during screening.

The following baseline measurements will be made during screening: Weekly mean VAS-Score, PGA-KOA, painDETECT, WOMAC, SF-36, 6 minute walking test.

Additionally a full medical history, physical examination, ECG, measurement of height and weight, and measurement of blood pressure will be performed during screening.

A safety laboratory will be performed at screening (V1) and at , V6, V9 and follow-up (See Table 1).

Safety Assessment Cannabidiol

A number of clinical studies investigating cannabidiol have been undertaken in two rare forms of epilepsia: Dravet Syndrom and Lennox-Gastaut Syndrom.

In a randomized, dose-ranging safety trial of cannabidiol in Dravet Syndrom [3] dosages of 5, 10 and 20mg/kg/d were generally well tolerated although adverse events (AE) were more common in the treatment arms than in the placebo arm. The most common AEs included pyrexia, somnolence, decreased appetite, sedation, ataxia and abnormal behavior. 22% of patients had an elevation of liver enzymes, ALT or AST > 3 X the upper limit of normal (ULN).

In a larger study on the effect of CBD on drop seizures in Lennox-Gastaut Syndrom, addition of CBD to a conventional antiepileptic regime at a dose of 10mg or 20mg/kg/d, CBD proved superior to placebo in reduction of frequency of seizures. AEs included elevated liver enzyme concentrations, somnolence, decreased appetite, diarrhea, pyrexia and vomiting. [5]

In an open-label extension trial of CBD in Dravet Syndrom 264 patients received a mean dose of 20mg/kg/d for a median of 274 days. The most common AEs were diarrhea, pyrexia, decreased appetite, and somnolence, and most were mild to moderate in severity.
The authors concluded that CBD was generally well tolerated and had an acceptable safety profile. [4]

	**Diagnosis**	**Dose**	**Age of Subjects**	**Duration**	**No. of Subjects**

Devinsky et al. Neurology, 2018	Dravet Syndrome	5, 10 or 20mg/kg/d	4-10 years	3 weeks treatment	34
Devinsky et al. NEJM, 2018	Lennox-Gastaut-Syndrome	10 or 20mg/kg/d	2-55 years	14 weeks	225
Devinsky et al. Epilepsia, 2018	Dravet Syndrome	2,5-20mg/kg/d	2-55 years	274 days median treatment duration	248

An oral solution of cannabidiol (Epidiolex®) was FDA-approved for the treatment of Lennox-Gastaut and Dravet-Syndrom in June 2018.

<u>Necessity of Patient Study-ID</u>

In Austria CBD is not a scheduled narcotic. It is freely available in Austrian retail and can be purchased legally. CBD is not tested for in any drug test panel in use. Therefore, a specific study ID aimed at avoiding legal consequences in case of a positive drug test is not warranted.

Study medication

<u>Cannabidiol (CBD)</u>

Active agent and characteristics:

crystalline CBD, isolated from hemp via solvent extraction, followed by flash chromatography and recrystallisation (BSPG Ltd., Sandwich, UK)

Trade name of the agent: Cannabidiol

Manufacturer: The CBD used as API has GMP-quality, is imported by TRIGAL GmbH, Vienna, and has a purity > 99.8%. THC-content is below the limit of detection (<0.01%). Other byproducts (cannabidivarin, butyl-derivative CBD) are of natural origin.

Capsules used in this study are prepared by Hubertus Apotheke, Spittal/Drau, Austria.

All steps follow GMP standards.

Drug supply: BSPG Laboratories

Storage Instructions:

Pure, crystalline CBD is stable >12 months at a temperature <25°C, if protected from light.

Capsules should be stored at ambient temperature (≤25°C), protected from light.

Stability data demonstrate a loss of CBD ≤0.126% per month.

Route of administration: Per os

Placebo

Ingredients: Fat, Ascorbyl Pamitate (E304), gelatine capsules (gelatine, titandioxide and/or other approved food colouring)

Manufacturer: Hubertus Apotheke, Spital/Drau, Austria

Storage: Capsules should be stored at ambient temperature (≤25°C), protected from light.

Route of administration: per os

Dosage and administration

Initial dose: 200mg/die

Maintenance dose: 600mg/die delivered in three doses per day (200mg-200mg-200mg)

Route of administration: oral administration

Duration: 9 weeks including titration period (1 week) and taper period (1 week)

Study-drug up- and down titration

Please see table 2. and table 3. for titration and taper scheme.

Capsules containing 200mg of CBD respectively will be utilized for titration and tapering.

During titration patients will receive 3 capsules morning, midday and evening.

During tapering patients will receive 3 capsule morning, midday and evening.

Study drug interruption or discontinuation

The Investigator must temporarily interrupt or permanently discontinue the study drug if continued administration of the study drug is believed to be contrary to the best interests of the patient.

The interruption or premature discontinuation of study drug might be triggered by an AE, a diagnostic or therapeutic procedure, an abnormal assessment (e.g., laboratory abnormalities), or for administrative reasons, in particular withdrawal of the patient's consent.

The reason for study drug interruption or premature permanent discontinuation must be documented in the CRF.

Study drug premature permanent discontinuation

Study drug premature permanent discontinuation due to an adverse event

If the reason for premature permanent discontinuation of study treatment is an AE, the patient will have a "Premature End of Study (EOS)" visit with all the assessments performed before the study drug discontinuation, whenever possible.

Study drug premature permanent discontinuation due to another reason than adverse event

If the reason for premature permanent discontinuation of study treatment is not an AE, the patient should be withdrawn from the study (withdrawal of consent) and have the end of study (EOS) visit with all the assessments performed before the study drug discontinuation, whenever possible.

Study-drug delivery & drug storage conditions

Capsules will be delivered by the manufacturer CBD 200mg and placebo.

Capsules should be stored at ambient temperature (≤25°C), protected from light.

Study drug packaging and labeling

Medication will be delivered to our clinic as verum and placebo.

Medication will be packaged in dispenser boxes with compartments for a morning and an evening dose by medical personnel at our clinic *not* involved in any of the study measures.

The boxes will be labeled with the study title, study center and contact, patient number, medication expiry date, storage conditions and directions for use.

For a sample label of study medication please see appendix.

IMP administration & handling

Capsules should be taken orally after a fatty meal to optimize absorption

Drug accountability

Drug accountability will be recorded at on-going basis on paper form (patient diary). Drug dispensing will be entered into the CRF. Furthermore, the correct intake of IMP or any variations concerning that will be recorded in the CRF at each visit during treatment period.

Procedures to assess subjects' compliance

Subject compliance will be monitored in form of a paper patient diary.

Concomitant medication

The well-being of the patient has the first priority, and modifications of concomitant treatment during the trial are allowed as necessary. They should be documented in the patient's records.

Allowed: All patients will receive paracetamol 3 x 1g PD as their basic analgesic medication.

Patients will be allowed tramadol drops (50mg up to 6 times per day) as rescue medication

Not allowed: opioids except for tramadol, benzodiazepines other than indicated at low doses for sleep disorders, **NSAID,** Corticosteroids (see also Exclusion Criteria).

Emergency procedure for unblinding

'Emergency' is defined as a Serious Adverse Event which is possibly related, probably related or related to the study drug.

A patient's treatment assignment should only be unblinded when knowledge of the treatment is essential to make a decision on the medical management of the patient.

In such a case the blinding table, which is held at our out-patient clinic, will be available at all times through the physician on duty.

Study procedures

General rules for trial procedures

- All study measures like blood sampling and measurements (vital parameters, ECG, etc.) have to be documented with date (dd:mm:yyyy).
- In case several study procedures are scheduled at the same time point, there is no specific sequence that should be followed.
- The dates of all procedures should be according to the protocol. The time margins mentioned in the study flow chart are admissible. If for any reason, a study procedure is not performed within scheduled margins a protocol deviation should be noted, and the procedure should be performed as soon as possible or as adequate.
- If it is necessary for organizational reasons, it is admissible to perform procedures which are scheduled for one visit at two different time points. Allowed time margins should thereby not be exceeded.

Screening investigation

Please see Table 1. Study Design/Visits

End-of-study (EOS) examination

After a treatment period of 8 weeks and a taper period of 1 week, patients undergo the end-of-study examination (Follow-Up) that entails:

WOMAC, PGA-KOA, mean weekly VAS-Score, ECG, physical examination, height and weight, blood pressure, safety laboratory, adverse events and blinding survey. (Please see table 1)

Definition of the end of the trial

The end of the trial is defined as the date of the last visit of the last patient undergoing the trial

SAFETY DEFINITIONS AND REPORTING REQUIREMENTS

Adverse events (AEs)

Summary of known and potential risks of the study drug

Please see safety assessment of Cannabidiol above.

Definition of adverse events

An AE is any untoward adverse change from the subject's baseline condition, i.e., any unfavourable and unintended sign including an abnormal laboratory finding, symptom or disease which is considered to be clinically relevant by the physician that occurs during the course of the study, whether or not considered related to the study drug.

Adverse events include:

- Exacerbation of a pre-existing disease.
- Increase in frequency or intensity of a pre-existing episodic disease or medical condition.
- Disease or medical condition detected or diagnosed after study drug administration even though it may have been present prior to the start of the study.
- Continuous persistent disease or symptoms present at baseline that worsen following the start of the study.
- Lack of efficacy in the acute treatment of a life-threatening disease.
- Events considered by the Investigator to be related to study-mandated procedures.
- Abnormal assessments, e.g., ECG and physical examination findings, must be reported as AEs if they represent a clinically significant finding that was not present at baseline or worsened during the course of the study.

- Laboratory test abnormalities must be reported as AEs if they represent a clinically significant finding, symptomatic or not, which was not present at baseline or worsened during the course of the study or led to dose reduction, interruption or permanent discontinuation of study drug.

Adverse events do not include:

- Pre-planned interventions or occurrence of endpoints specified in the study protocol are not considered AE´s, if not defined otherwise (eg.as a result of overdose)
- Medical or surgical procedure, e.g., surgery, endoscopy, tooth extraction, transfusion. However, the event leading to the procedure is an AE. If this event is serious, the procedure must be described in the SAE narrative.
- Pre-existing disease or medical condition that does not worsen.
- Situations in which an adverse change did not occur, e.g., hospitalizations for cosmetic elective surgery or for social and/or convenience reasons.
- Overdose of either study drug or concomitant medication without any signs or symptoms. However, overdose must be mentioned in the Study Drug Log.

Serious adverse events (SAEs)

A Serious Adverse Event (SAE) is defined by the International Conference on Harmonization (ICH) guidelines and GCP guidelines as any AE fulfilling at least one of the following criteria:

- Results in deaths.
- Life-threatening – defined as an event in which the subject was, in the judgment of the Investigator, at risk of death at the time of the event;
- Requiring subject's hospitalization or prolongation of existing hospitalization
- Resulting in persistent or significant disability or incapacity (i.e., a substantial disruption of a person’s ability to conduct normal life functions).
- Congenital anomaly or birth defect.
- Optional: Is medically significant or requires intervention to prevent at least one of the outcomes listed above

Life-threatening refers to an event in which the subject was at risk of death at the time of the event. It does not refer to an event that hypothetically might have caused death if it were more severe.

Important medical events that may not immediately result in death, be life-threatening, or require hospitalization may be considered as SAEs when, based upon appropriate medical judgment, they may jeopardize the subject and may require medical or surgical intervention to prevent one of the outcomes listed in the definitions above. This means an individual case decision.

Hospitalization – Prolongation of existing hospitalization

Hospitalization is defined as an overnight stay in a hospital unit and/or emergency room.

An additional overnight stay defines a prolongation of existing hospitalization.

The following is not considered an SAE and should be reported as an AE only:

- Treatment on an emergency or out subject basis for an event not fulfilling the definition of seriousness given above and not resulting in hospitalization.

The following reasons for hospitalizations are not considered AEs, and therefore not SAEs:

- Hospitalizations for cosmetic elective surgery, social and/or convenience reasons.
- Elective treatment of a pre-existing disease or medical condition that did not worsen, e.g., hospitalization for chemotherapy for cancer, elective hip replacement for arthritis.

SAEs related to investigational drug

Such SAEs are defined as SAEs that appear to have a reasonable possibility of causal relationship.

Suspected unexpected serious adverse reactions (SUSARs)

SUSARs are all serious adverse reactions with **suspected** causal relationship to the study drug that is **unexpected** (not previously described in the Summary of Product Characteristics or Investigator's brochure) and serious.

Pregnancy

Any pregnancy that occurs during study participation must be reported to the Investigator/sponsor. To ensure subject safety, each pregnancy must be reported to the Sponsor immediately. The pregnancy must be followed up to determine outcome (including premature termination) and status of mother and child. Pregnancy complications and elective terminations for medical reasons must be reported as an AE or SAE. Spontaneous abortions must be reported as an SAE.

Any SAE occurring in association with a pregnancy brought to the Investigator's attention after the subject has completed the study and considered by the Investigator as possibly related to the investigational product, must be promptly reported to the Investigator/sponsor.

In addition, the Investigator must attempt to collect pregnancy information on any female partners of male study subjects who become pregnant while the subject is enrolled in the study. Pregnancy information must be reported to the Investigator/sponsor as described above.

Severity of adverse events

The severity of clinical AEs is graded on a three-point scale: mild, moderate, severe, and reported on specific AE pages of the CRF.

If the severity of an AE worsens during study drug administration, only the worst intensity should be reported on the AE page. If the AE lessens in intensity, no change in the severity is required.

If an AE occurs during a washout or placebo run-in phase and afterwards worsens during the treatment phase, a new AE page must be filled in with the intensity observed during study drug administration.

Mild

Event may be noticeable to subject; does not influence daily activities; the AE resolves spontaneously or may require minimal therapeutic intervention;

Moderate

Event may make subject uncomfortable; performance of daily activities may be influenced; intervention may be needed; the AE produces no sequelae.

Severe

Event may cause noticeable discomfort; usually interferes with daily activities; subject may not be able to continue in the study; the AE produces sequelae, which require prolonged therapeutic intervention.

A mild, moderate or severe AE may or may not be serious. These terms are used to describe the intensity of a specific event (as in mild, moderate, or severe myocardial infarction). However, a severe event may be of relatively minor medical significance (such as severe headache) and is not necessarily serious. For example, nausea lasting several hours may be rated as severe, but may not be clinically serious. Fever of 39°C that is not considered severe may become serious if it prolongs hospital discharge by a day. Seriousness rather than severity serves as a guide for defining regulatory reporting obligations.

Relationship to study drug

For all AEs, the Investigator will assess the causal relationship between the study drug and the AE using his/her clinical expertise and judgment according to the following algorithm that best fits the circumstances of the AE:

Not related

- May or may not follow a temporal sequence from administration of the study product
- Is biologically implausible and does not follow known response pattern to the suspect study drug (if response pattern is previously known).
- Can be explained by the known characteristics of the subject's clinical state or other modes of therapy administered to the subject.

Unlikely

- There is a reasonable temporal relation between the AE and the intake of the study medication, but there is a plausible other explanation for the occurrence of the AE.

Possible

- The AE has a reasonable temporal relationship with drug administration.
- The AE may equally be explained by the study subject's clinically state, environmental or toxic factors, or concomitant therapy administered to the study subject.
- The relationship between study drug and AE may also be pharmacologically or clinically plausible.

Probable

- There is a reasonable temporal relation between the AE and the intake of the study medication, and plausible reasons point to a causal relation with the study medication.

Related

- Reasonable temporal relation between the AE and the intake of the study medication and

- There is no other explanation for the AE and
- Subsidence or disappearance of the AE on withdrawal of the study medication and
- Recurrence of the symptoms on restart at previous dose (only applies for re-institution of mediation).

Not assessable

- The causal relationship between the study drug and the AE cannot be judged.

Reporting procedures

A special section is designated to adverse events in the case report form. The following details must thereby be entered:

- Type of adverse event
- Start (date and time)
- End (date and time)
- Severity (mild, moderate, severe)
- Serious (no / yes)
- Unexpected (no / yes)
- Outcome (resolved, resolving, not resolved, resolved with sequelae, unknown, fatal)
- Relation to study drug (Related/ Probably/ Possibly/ Unlikely/ Not related/ Not assessable)

Adverse events are to be documented in the case report form in accordance with the above mentioned criteria.

Reporting procedures for SAEs

In case of a serious adverse event, the Investigator has to use all supportive measures for best patient treatment. A written report is also to be prepared and should at least contain the following:

- Patient number
- Patient: sex
- The suspected investigational medical product (IMP)
- The adverse event assessed as serious
- Short description of the event and outcome

If applicable, the initial report should be followed by the Follow up report, indicating the outcome of the SAE.

Reporting procedures for SUSAR

It must be remembered that the regulatory authorities, and the Institutional Review Board / Independent Ethics Committee (IRB / IEC) must be informed about all SUSAR. Such reports shall be made by the sponsor and should content at least the following details:

- Patient number (study code/screening number)
- Patient: age in years, sex
- Name of Investigator and investigating site
- Period of administration

- The suspected investigational medical product (IMP)
- The adverse event assessed as serious and unexpected, and for which there is a **suspected** causal relationship to the IMP
- Concomitant disease and medication
- Short description of the event:
    - Description
    - Onset and if applicable, end
    - Therapeutic intervention
    - Causal relationship
    - Seriousness criteria or reportable reason

Electronic reporting should be the expected method for reporting of SUSARs to the competent authority. In that case, the format and content as defined by the regulatory requirements should be adhered to. The latest version of MedDRA should be applied. Lower-level terms (LLT) should be used.

DOCUMENTATION AND DATA MANAGEMENT

Documentation of study results

A subject screening and identification Log will be completed for all enrolled subjects with the reasons for exclusion.

Case report form (CRF)

In the current study paper based CRFs will be used.

For each subject enrolled, regardless of study drug initiation, a CRF must be completed and signed by the Investigator or a designated sub-Investigator. This also applies to those subjects who fail to complete the study. If a subject withdraws from the study, the reason must be noted on the CRF. Case report forms are to be completed on an ongoing basis.

If screening failures should not be documented in the CRF, this has to be clearly defined in the protocol.

CRF entries and corrections will only be performed by study site staff, authorized by the Investigator.

In a paper based CRF all forms should be completed and must be legible. Entry errors have to be corrected according the ICH-GCP Guidelines.

The entries will be checked by trained personnel (Monitor) and any errors or inconsistencies will be checked immediately.

The monitor will collect original completed and signed CRFs at the end of the study. The completed and signed CRFs will remain on site.

Data Manager: Dr. Regina Patricia Schukro,

Department of Cardiothoracic and Vascular Anaesthesia and Intensive Care Medicine, Medical University of Vienna

Regina.schukro@meduniwien.ac.at

Data collection

Data collected at all visits are entered into a paper CRF. The CRFs will be source documents verified following guidelines established before study onset as detailed in the Monitoring Plan.

Safekeeping

The Investigator will maintain adequate and accurate records to enable the conduct of the study to be fully documented and the study data to be subsequently verified (according to ICH-GCP "essential documents"). These documents will be classified into two different categories: Investigator's study site file (ISF) with all essential documents regarding the study conduct, and subject clinical source documents.

The Investigator's file will contain all essential documents listed in ICH-GCP Guidelines section 8.

Subject clinical source documents include all patient hospital clinical records in original version, such as original laboratory reports, ECG, X-ray prints and other reports.

These two categories of documents must be kept on file by the Investigator for as long as needed to comply with the regulatory requirements.

Quality control and quality assurance

Periodic Monitoring

The designated monitor will contact and visit the Investigator on a regularly basis and will be allowed to have direct access to all source documents needed to verify the entries in the CRFs and other protocol-related documents provided that subject confidentiality is maintained in agreement with local regulations. It will be the monitor's responsibility to inspect the CRFs at regular intervals according to the monitoring plan throughout the study, to verify the adherence to the protocol and the completeness, consistency and accuracy of the data being entered on them.

Monitoring will be performed by Ao. Prof. Dr. Andrea Michalek-Sauberer,

andrea.michalek-sauberer@meduniwien.ac.at

Department of Anesthesiology and Pain Medicine, Medical University Vienna

4 monitoring visits are scheduled (initiation visit, three routine visits (every 6 months) and a close out visit after the last patient has completed the study)

The monitor will check the source data on the following points for each patient:

1) Informed Consent

2) Adverse Events

3) Primary Endpoint

100% of source data will be inspected in a control sample.

Audit and inspections

Upon request, the Investigator will make all study-related source data and records available to a qualified quality assurance auditor mandated by the sponsor or to competent authority inspectors. The main purposes of an audit or inspection are to confirm that the rights and welfare of the subjects have been adequately protected, and that all data relevant for assessment of safety and efficacy of the investigational product have appropriately been reported to the sponsor.

Reporting and publication

Publication of study results

The findings of this study will be published by the sponsor (Investigators) in a scientific journal and presented at scientific meetings. The manuscript will be circulated to all co-Investigators before submission. Confidentiality of subjects in reports/publications will be guaranteed.

ETHICAL AND LEGAL ASPECTS

Informed consent of subjects

Following comprehensive instruction regarding the nature, significance, impact and risks of this clinical trial, the patient must give written consent to participation in the study.

During the instruction the trial participants are to be made aware of the fact that they can withdraw their consent – without giving reasons – at any time without their further medical care being influenced in any way.

In addition to the comprehensive instructions given to the trial participants by the Investigator, the trial participants also receive a written patient information sheet in comprehensible language, explaining the nature and purpose of the study and its progress.

The patients must agree to the possibility of study-related data being passed on to relevant authorities.

The patients must be informed in detail of their obligations in relation to the trial participants insurance in order not to jeopardize insurance cover.

Acknowledgement / approval of the study

The Investigator (or a designated CRO) will submit this protocol and any related document provided to the subject (such as subject information used to obtain informed consent) to an Ethics Committee (EC) or Institutional Review Board (IRB). Approval from the committee must be obtained before starting the study.

The clinical trial shall be performed in full compliance with the legal regulations according to the Drug Law (AMG - Arzneimittelgesetz) of the Republic of Austria.

An application must also be submitted to the Austrian Competent Authorities (Bundesamt für Sicherheit im Gesundheitswesen (BASG) represented by the Agency for Health and Food Safety (AGES Medizinmarktaufsicht) and registered to the European Clinical Trial Database (EudraCT) using the required forms. The timelines for (silent) approval set by national law must be followed before starting the study.

Changes in the conduct of the study

Protocol amendments

Proposed amendments must be submitted to the appropriate CA and ECs. Substantial amendments may be implemented only after CA/EC approval has been obtained. Amendments that are intended to eliminate an apparent immediate hazard to subjects may be implemented prior to receiving CA/EC approval. However, in this case, approval must be obtained as soon as possible after implementation.

Study Termination

If the sponsor or the Investigator decides to terminate the study before the planned completion, they will notify each other in writing stating the reasons of early termination. Both the sponsor and the investigator will ensure the protection of the subjects' wellbeing. The sponsor will notify the regulatory authority as well as the ethics committee about the premature termination. Documentation will be filed in the Trial Master File as well as in the Investigator Site File.

Clinical Study Report (CSR)

Within one year after the final completion of the study, a full CSR will be prepared by the sponsor and submitted to the EC and the competent authority.

The Investigator will be asked to review and sign the final study report.

Insurance

During their participation in the clinical trial the patients will be insured as defined by legal requirements. The Investigator of the clinical trial will receive a copy of the insurance conditions of the 'patients' insurance'. The sponsor is providing insurance in order to indemnify (legal and financial coverage) the Investigator/centre against claims arising from the study, except for claims that arise from malpractice and/or negligence. The compensation of the subject in the event of study-related injuries will comply with the applicable regulations.

Details on the existing patients insurance are given in the patient information sheet.

All subjects participating in clinical studies will be insured through the Department of Anaesthesiology and Pain Medicine (master policy, Medical University Vienna) by:

Zürich Versicherungs AG

Address: Schwarzenbergplatz 15, 1010, Wien

Telephonnumber: 0043 (01) 50125-0

Insurance Policy Number: 07229622-2

Ethics and good clinical practice (GCP)

The Investigator will ensure that this study is conducted in full conformance with the principles of the "Declaration of Helsinki" (as amended at the 64th WMA General Assembly, Fortaleza, Brazil, 2013) and with the laws and regulations of the country in which the clinical research is conducted.

The Investigator of the clinical trial shall guarantee that only appropriately trained personnel will be involved in the study. All studies must follow the ICH GCP Guidelines and the regulatory requirements.

Therefore, this study follows the EU Directive embedded in the Austrian drug act

- **Potential inconveniences and risks for the patients**

Drug

Patients will be informed about all potential side effects of the administered drug cannabidiol.

A detailed safety evaluation is presented above.

Blood loss

The total blood loss amounts to 46 ml consisting of blood sampling over a period of 13 weeks.

APPENDIX

Sample Label Study Medication

Studienmedikation:
Wirksamkeit von Cannabidiol in der Behandlung von Schmerzen bei symptomatischer Arthrose des Kniegelenks. Eine randomisiert doppelblinde placebo-kontrollierte Studie

Durchführende Stelle:
Klinische Abteilung für spezielle Anästhesie und Schmerzmedizin, Medizinische Universität Wien, Währingergürtel 18-20, 1090, Wien

Kontakt:
Dr. Sibylle Pramhas
Ständig erreichbar unter: +436507062112
Dr. Mirhosseini-Vakili, Mohammed Ali
Ständig erreichbar unter: +43 699 17000144

Patientennummer: XX

Ablaufdatum:
Bekanntgegeben durch Hersteller

Lagerungshinweise:
Bei unter 25°C, dunkel lagern.
Außerhalb der Reichweite von Kindern aufbewahren

Einnahme:
Zur mündlichen Einnahme

References

1. O'Neill TW, McCabe PS, McBeth J: Update on the epidemiology, risk factors and disease outcomes of osteoarthritis. Best Pract Res Clin Rheumatol 2018; 32: 312-326
2. Fasinu PS, Phillips S, ElSohly MA, Walker LA: Current Status and Prospects for Cannabidiol Preparations as New Therapeutic Agents. Pharmacotherapy 2016; 36: 781-96
3. Devinsky O, Patel AD, Thiele EA, Wong MH, Appleton R, Harden CL, Greenwood S, Morrison G, Sommerville K, Group GPAS: Randomized, dose-ranging safety trial of cannabidiol in Dravet syndrome. Neurology 2018; 90: e1204-e1211
4. Devinsky O, Nabbout R, Miller I, Laux L, Zolnowska M, Wright S, Roberts C: Long-term cannabidiol treatment in patients with Dravet syndrome: An open-label extension trial. Epilepsia 2018
5. Devinsky O, Patel AD, Cross JH, Villanueva V, Wirrell EC, Privitera M, Greenwood SM, Roberts C, Checketts D, VanLandingham KE, Zuberi SM, Group GS: Effect of Cannabidiol on Drop Seizures in the Lennox-Gastaut Syndrome. N Engl J Med 2018; 378: 1888-1897
6. Ribeiro A, Almeida VI, Costola-de-Souza C, Ferraz-de-Paula V, Pinheiro ML, Vitoretti LB, Gimenes-Junior JA, Akamine AT, Crippa JA, Tavares-de-Lima W, Palermo-Neto J: Cannabidiol improves lung function and inflammation in mice submitted to LPS-induced acute lung injury. Immunopharmacol Immunotoxicol 2015; 37: 35-41
7. Li K, Feng JY, Li YY, Yuece B, Lin XH, Yu LY, Li YN, Feng YJ, Storr M: Anti-inflammatory role of cannabidiol and O-1602 in cerulein-induced acute pancreatitis in mice. Pancreas 2013; 42: 123-9
8. Philpott HT, O'Brien M, McDougall JJ: Attenuation of early phase inflammation by cannabidiol prevents pain and nerve damage in rat osteoarthritis. Pain 2017; 158: 2442-2451
9. Sumariwalla PF, Gallily R, Tchilibon S, Fride E, Mechoulam R, Feldmann M: A novel synthetic, nonpsychoactive cannabinoid acid (HU-320) with antiinflammatory properties in murine collagen-induced arthritis. Arthritis Rheum 2004; 50: 985-98
10. Xiong W, Cui T, Cheng K, Yang F, Chen SR, Willenbring D, Guan Y, Pan HL, Ren K, Xu Y, Zhang L: Cannabinoids suppress inflammatory and neuropathic pain by targeting alpha3 glycine receptors. J Exp Med 2012; 209: 1121-34
11. Costa B, Trovato AE, Comelli F, Giagnoni G, Colleoni M: The non-psychoactive cannabis constituent cannabidiol is an orally effective therapeutic agent in rat chronic inflammatory and neuropathic pain. Eur J Pharmacol 2007; 556: 75-83
12. Aviram J, Samuelly-Leichtag G: Efficacy of Cannabis-Based Medicines for Pain Management: A Systematic Review and Meta-Analysis of Randomized Controlled Trials. Pain Physician 2017; 20: E755-E796
13. Altman R, Asch E, Bloch D, Bole G, Borenstein D, Brandt K, Christy W, Cooke TD, Greenwald R, Hochberg M, et al.: Development of criteria for the classification and reporting of osteoarthritis. Classification of osteoarthritis of the knee. Diagnostic and Therapeutic Criteria Committee of the American Rheumatism Association. Arthritis Rheum 1986; 29: 1039-49
14. Conaghan PG, Dickson J, Bolten W, Cevc G, Rother M: A multicentre, randomized, placebo- and active-controlled trial comparing the efficacy and safety of topical ketoprofen in Transfersome gel (IDEA-033) with ketoprofen-free vehicle (TDT 064) and oral celecoxib for knee pain associated with osteoarthritis. Rheumatology (Oxford) 2013; 52: 1303-12
